# High-Resolution Lipidomics Reveals Influence of Biomass and Pretreatment Process on the Composition of Extracted Algae Oils As Feedstock for Sustainable Aviation Fuels

**DOI:** 10.1021/acs.energyfuels.3c04857

**Published:** 2024-03-27

**Authors:** Steven M. Rowland, Stefanie Van Wychen, Tao Dong, Roger Leach, Lieve M. L. Laurens

**Affiliations:** † Bioenergy Science and Technology Directorate, National Renewable Energy Laboratory, 15013 Denver West Parkway, Golden, Colorado 80401, United States; ‡ Viridos (formerly Synthetic Genomics), 11149 N Torrey Pines Road, La Jolla, California 92037, United States

## Abstract

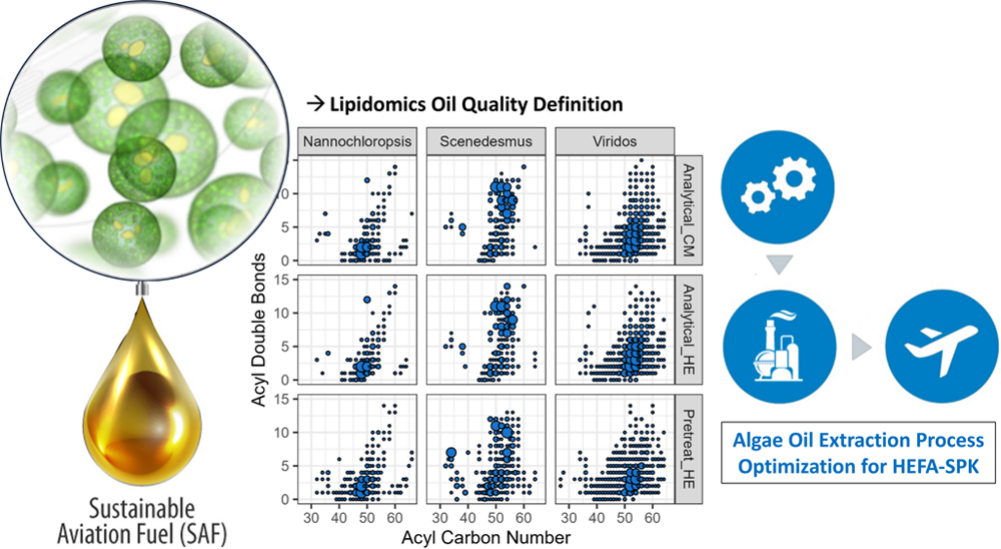

The increasing demand for sustainable aviation fuel (SAF) creates a need for innovative biomass and lipid sources with compositions that are compatible with refineries. Algae-derived oils present an opportunity to supply a process-compatible lipid feedstock at yields higher than those of conventional oilseed crops. With few documented reports on chemical composition, the process readiness remains elusive. We present data on extraction efficiency, yield, and purity of lipids from algae with and without the application of a low-concentration sulfuric acid pretreatment of the biomass. The pretreatment process increased the oil yield and positively impacted the quality of the extracted oils. Results from fatty acid and lipidomics analysis revealed that the low-lipid biomass sources extracted 70–80% of the available lipids, and the non-fatty acid co-extractants exceeded 40% of the extracted oils. For a high-lipid algae sample, derived from a genetically engineered strain, we show >90% extraction yield with >85% FAME purity. This work provides insights into the composition of algae-derived oils and quality metrics that are essential to determining the viability of lipid hydroprocessing to SAF.

## Introduction

The demand for sustainable aviation fuel (SAF) is rapidly increasing, necessitating a diverse portfolio of feedstock types to produce adequate volumes required for commercial aviation demand.^1^ The current limits on the availability of conventional feedstocks such as soybean oil, distillers corn oil, and other fats, oils, and greases (FOGs) for hydrotreated esters and fatty acids (HEFA) synthetic paraffinic kerosene (SPK) upgrading, indicate challenges in meeting the target volumetric requirements.^1,2^ In this context, algae have long been included as an attractive option to contribute to an expanded portfolio of feedstocks, thanks to high yields, ability to produce on non-arable lands, using wastewater and seawater, and grow in arid environments, with an intrinsic high affinity for photosynthetic CO_2_ capture.^3,4^

Currently there is an ASTM approved pathway from fats, oils, and greases (FOGs) to SAF via hydroprocessing in the well-documented HEFA-SPK pathway.^5^ Conventional feedstocks typically comprise vegetable oils, palm oil, used cooking oil, animal fats, and other fatty acid distillates, which all have been included as feedstocks into SAF biorefineries, with the resulting SAF blended up to 50% into aviation fuel formulations. The specifications for HEFA-SPK feedstocks define the necessary oil clean up steps and include metrics around free fatty acid content (*e.g.*, <10% by weight for vegetable oils and used cooking oils but larger contributions allowed for animal fats, up to 35%), phosphorus (<250 ppm for most vegetable oils, and up to 1,000 ppm for animal fats), metals (similar to P requirements), nitrogen (<200 ppm for vegetable oils and up to 1,500 ppm for animal fats), sulfur (<100 ppm for most fats and oils, with <200 for animal fats), chlorine (<15 ppm for vegetable oils, with up to 100 ppm for used cooking oils and up to 500 ppm used for animal fats).^6^ Each of the feedstocks will necessitate a custom configuration of oil pretreatment (*e.g.*, a combination of filtration, acid degumming, bleaching and additional heat treatment).^6^ For novel feedstocks, like algae oils, while successful hydrotreating has been demonstrated,^7^ the chemical composition of oils derived from a range of different algae origins is not well characterized, and thus hydrotreating-compatibility questions can slow the possible adoption into existing refineries.^8^ It is anticipated that the quality of algae oils will fall in between the quality of pristine vegetable oils and animal fats and thus necessitate further oil treatment prior to biorefinery intake.

The HEFA-SPK pathway is often assumed to be fully compatible with algae-derived lipids and oils. Prior work has demonstrated the utility of ultrahigh resolution mass spectrometry for the analysis of triglycerides, polar lipids and pigments derived from microalgae.^9,10^ Because there is currently little information about the chemical composition of relevant oils extracted from algae and specifically how the oil composition changes with species and growth conditions, existing refineries are hesitant to accept, or plan for, algae oils.

The objective of this work was to provide an analytical framework to define the compositional profile of oils extracted from different algae-derived biomass samples in the context of compatibility with existing lipid feedstocks. We investigated oil extraction from three species of algae to determine the impact of biomass pretreatment and the extraction approach on the oil quality from a range of commercially relevant algae species. We applied ultrahigh-resolution mass spectrometry to determine the lipid composition for each oil based on accurate mass chemical formula assignment and ion-fragmentation. The lipidomics analysis was used to understand the impact of the pretreatment and extraction approach on the composition of extracted oils. Our work highlights that algae can be a good source of lipids for conversion to SAF, but that algae species, biomass composition, and choice of extraction method impact the overall quality of algae oil. A dilute sulfuric acid biomass pretreatment method, based on previous work,^11,12^ can be used to reduce the amount of nitrogen and phosphorus-containing lipids in algae oils from high-protein biomass, while extracting greater than 70% of the fatty acid material from the biomass. When high-lipid algal biomass is used in this process, the resulting oils align compositionally more closely to conventional vegetable oil feedstocks due to the inherently low heteroatom content and high extraction yields. We focus the molecular identification on complex N-containing lipid constituents and helping to elucidate not just oil quality and purification needs, but also the origin and fate of photosynthetic pigments. This approach provides key insights into the molecular composition of algae-derived oils and associated quality metrics for product specifications characterization, that is complementary to standard ASTM and AOCS methods that are available for the analysis of, *e.g.*, free fatty acid content, inorganics, and nitrogen content. The combined reporting of oil product specifications with molecular speciation will aid in understanding the viability of upgrading algae oil to SAF through to hydroprocessing.

## Results and Discussion

### Biomass Composition

To specifically study the impact of differences in biomass composition on the quality and yield of extractable oils, we created a sample set that is representative of common algae biomass that is currently being produced at scale. Two samples, *Nannochloropsis* (marine representative) and *Scenedesmus* (fresh/brackish-water representative), represent biomass from production operations that focus on rapid growth for high-value intermediate products (*e.g.*, *Nannochloropsis* for the production of polyunsaturated fatty acids), and their biochemical composition is dominated by high protein content, with lower carbohydrate and lipid content (<15% of the biomass each). A third sample was obtained from a collaborator, Viridos, Inc., representing a genetically engineered, high-lipid, marine microalgae (referred to as Strain #15), produced at large-scale at a facility near Calipatria, California. Table 1 illustrates the composition for each of the three samples as measured (and reported previously).^13^

**Table 1:** Biomass Composition for Microalgae Species on a Dry Basis, As Previously Described^a^^,^^13^

Species	Ash	Carbohydrates	Protein	FAME
*Scenedesmus* sp.	17.8 ± 0.2	9.0 ± 0.3	35.7 ± 0.8	5.4 ± 0.0
*Nannochloropsis* sp.	17.6 ± 0.1	8.3 ± 0.4	31.6 ± 0.5	9.6 ± 0.1
Viridos	35.0 ± 0.2	14.4 ± 0.1	5.9 ± 0.5	22.3 ± 1.8

a FAME = fatty acid methyl ester as a proxy for total lipid content through direct, *in situ*, transesterification. Data presented are mean ± standard deviation of at least duplicate measurements of freeze-dried biomass chemical composition.

### Effect of Solvent and Pretreatment on Lipid Recovery

**Figure 1:**
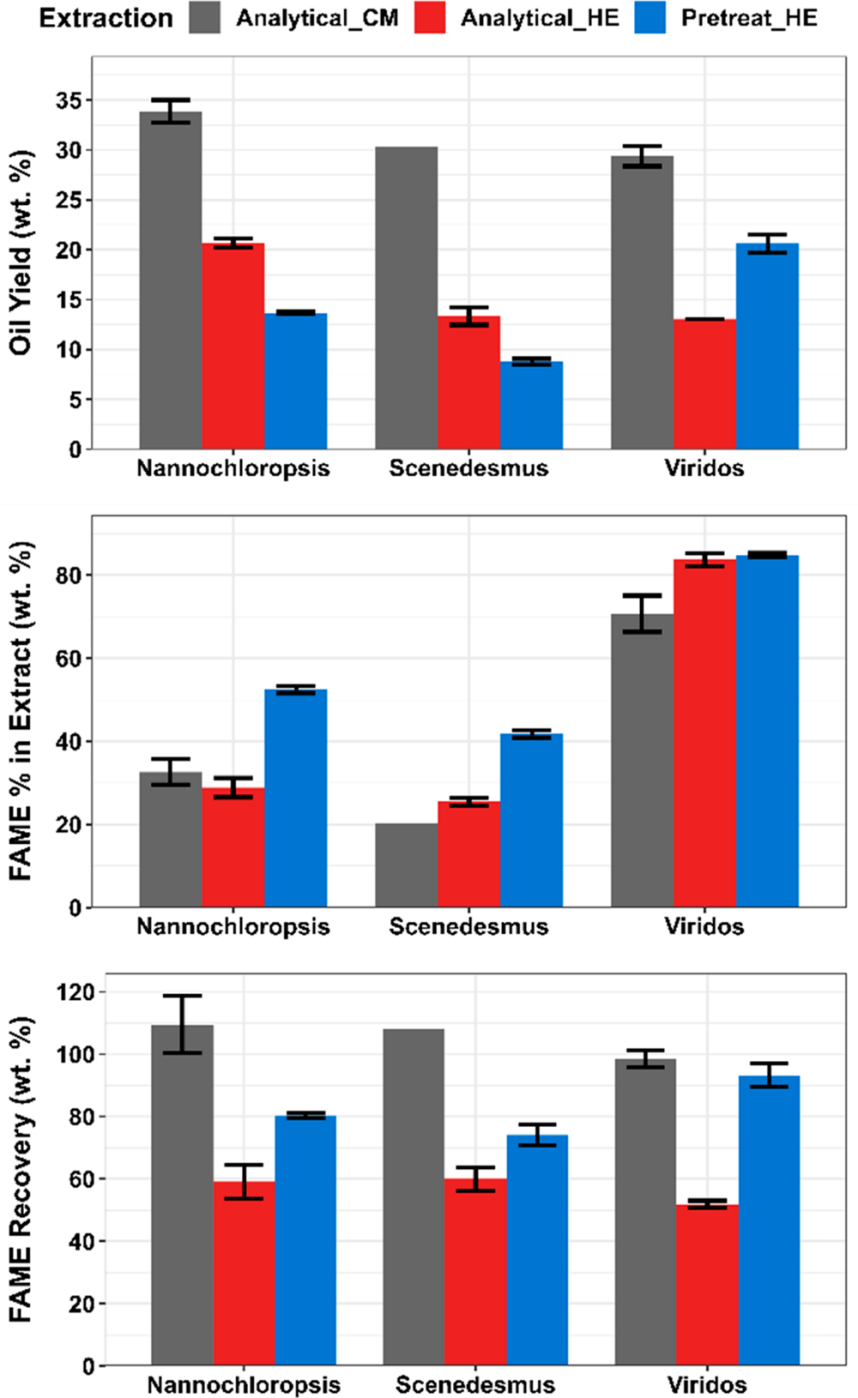
Extractable oil yield, FAME purity, and FAME yield data from analytical extractions with C:M and H:E and pretreatment extraction with H:E for *Nannochloropsis* sp., *Scenedesmus* sp., and a Viridos engineered algae strain. (Top) Mass yield of extracted oil on a dry biomass basis; (Middle) percentage of extracted oil that is identified by FAME analysis; and (Bottom) mass recovery of FAME in extracted oils on a biomass basis.

To test the effect of solvent on the yield and chemical composition of the extracted oils, two common solvent mixtures (chloroform:methanol, C:M, and hexane:ethanol, H:E, as representatives of typical analytical extractions)^13,14^ combined with mechanical cell disruption (bead beating) were compared to an industrially relevant dilute-acid pretreatment method.^11,12,15^ We observed that the C:M extraction resulted in the highest mass yield of extracted oil, measured as gravimetric extractable oils, compared to the other two approaches, which is consistent with the ubiquitous use of C:M as the reference total intact lipid extraction solvent system.^16,17^ However, it should be pointed out that the extracted mass for *Scenedesmus* and *Nannochloropsis* with C:M yielded 3- to 6-fold higher amounts than the initial FAME. This indicates that the selectivity of C/M toward lipids is poor, which results in coextracted materials. The analytical extraction with H:E, in all cases, yielded approximately 30–60% of the mass extracted with C:M (Figure 1), and the pretreated high-protein samples used in this work resulted in even lower extracted oil yield, possibly pointing to solvent mass transfer interferences due to the complex amphiphilic nature of the biomass.^18^ In contrast, the pretreatment of the high-lipid biomass sample (Viridos) showed an increased oil yield relative to the analytical extraction with H:E (Figure 1).

As a metric of oil purity, we routinely measure the total fatty acid content (as FAME) in extractable oils, and the higher the fraction of fatty acids, the higher is the predicted conversion or hydrotreating yield. We observed that all of the oils from acid-pretreated biomass showed significantly higher percentages of FAME in the extracted oils when compared to the analytical extractions with C:M and H:E. This result indicates that, although the mass yield for the pretreatment-extracted oils is lower for the high-protein biomass samples, there is a fatty acid enrichment effect that can be attributed to the pretreatment step, likely due to a decrease in coextracted non-lipid material. The FAME recovery (eq 1) showed that the analytical extraction with C:M recovered 100% of the FAME in the biomass; however, the oil was only composed of approximately 33 and 20% FAME for the *Nannochloropsis* and *Scenedesmus* samples, respectively, which indicates that most of the extracted components are not fatty acids and may be composed of compounds that are not likely to generate SAF. The high-lipid biomass sample showed that although the gravimetric extraction yield for the acid pretreatment was significantly lower when compared to the analytical C:M method, the extractable oil contained more than 80% of the FAME in the biomass. This, again, shows the improved selectivity of solvents like H/E in comparison to more polar solvents like C:M and suggests that efficient lipid recovery with acid pretreatment is feasible. This data also suggests that the fraction of undesirable coextractants is significantly reduced compared to conventional analytical extractions that utilized bead beating for cell disruption (Figure 1).
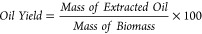
1
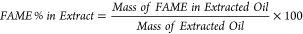
2
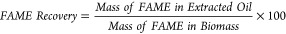
3

### Effect of Solvent and Pretreatment on Lipid Composition

**Figure 2:**
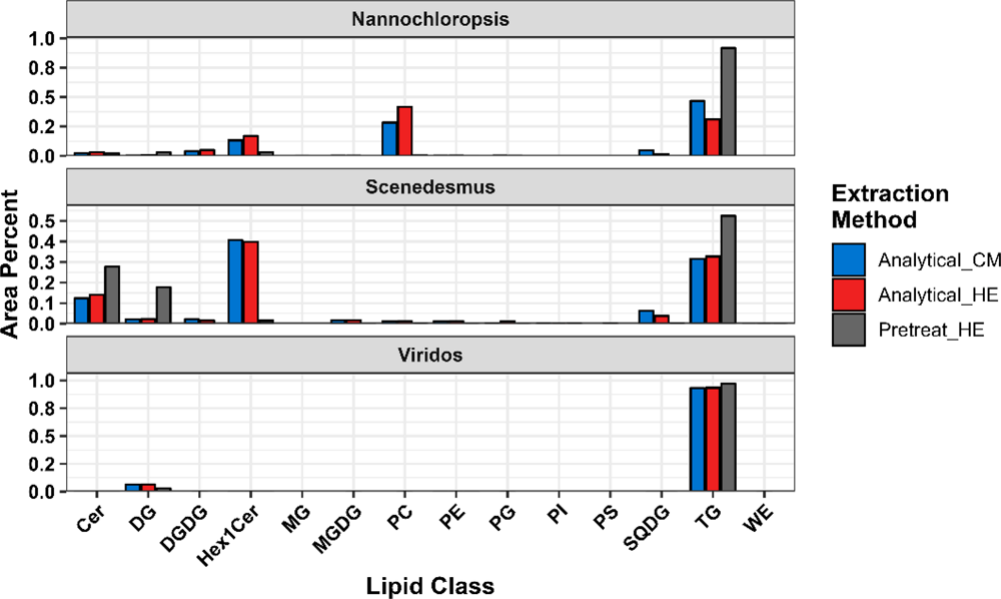
Lipid class graph from LC-MS-derived lipidomics analysis; data shown as a percentage of the total peak area of annotated lipids. Lipid abbreviations: Cer – ceramide, DG – diacylglycerol, DGDG – digalactosyldiacylglycerol, Hex1Cer – hexosylceramide, MGDG – monogalactosyldiacylglycerol, PC – phosphatidylcholine, PE – phosphatidylethanolamine, PG – phosphatidylglycerol, PI – phosphatidylinositol, PS – phosphatidylserine, SQDG – sulfoquinovosyldiacylglycerol, TG – triacylglycerol, WE – wax ester (lipidomics annotations are included in the Supporting Information).

To more deeply investigate the effect of the extraction and pretreatment methods on the chemical molecular composition, we carried out positive-ion lipidomics analysis by high-resolution mass spectrometry coupled to reversed phase chromatography. Lipidomics analysis shows the effects of solvent selection and pretreatment on the respective compositions of extracted oils. Figure 2 shows the lipid class graphs for the three algae oils based on the extraction procedure. We observed very little difference in the composition of the oils extracted with the analytical C:M and H:E methods, almost identical for *Scenedesmus* and only small changes observed in *Nannochloropsis*. However, the pretreated oils from high protein algae show significant reduction in polar lipids, including phospholipids, glycolipids, and hexosylceramides. Specifically, the removal of phosphorus in extracted oil is incredibly important as phosphorus from phospholipids has been identified as one of the most deleterious catalyst poisons for renewable oils.^19,20^ We did observe an increase in the ceramides detected in *Scenedesmus* after pretreatment, which is likely due to the cleavage of the hexose portion of the Hex1Cer lipids to produce higher concentrations of ceramides. Along with a decrease in polar lipids, we also observed an increase in the nonpolar TG and DG lipids for high protein algae with acid pretreatment. These results corroborate our observations from Figure 1 and show that the majority of the head groups of polar lipids are cleaved during acid pretreatment, which results in oil that is higher in the desired nonpolar lipids (i.e., higher FAME% in the oils). The high-lipid Viridos algae showed no appreciable change in the lipid composition, since the lipids produced by this strain were almost exclusively TG and DG lipids. This is an important observation, as it confirms that nonpolar lipids are not hydrolyzed or oxidized during dilute acid pretreatment. Figure S1 shows the acyl-carbon number vs acyl-double bonds for TG lipids for each sample and extraction condition. We observe that there is no major shift in the composition of nonpolar lipids, as indicated by the plots of acyl double bonds vs acyl carbons. The *Scenedesmus* sample does show a small increase in the more saturated TG lipids (i.e., 2–4 double bonds) compared to the more unsaturated lipids (i.e., greater than 7 double bonds) with acid pretreatment; however, considering that the extraction efficiency increases for TG lipids, this change could be due to better extraction of more saturated lipids with acid pretreatment. FAME profiles were also investigated in Figure S2, where the FAME of the extracted oils is plotted for all extractions and algae samples. We observe very little change in the FAME profiles due to extraction type. However, the *Scenedesmus* sample does show slightly greater variability compared to the other samples.

### Recalcitrant N Compounds

**Figure 3:**
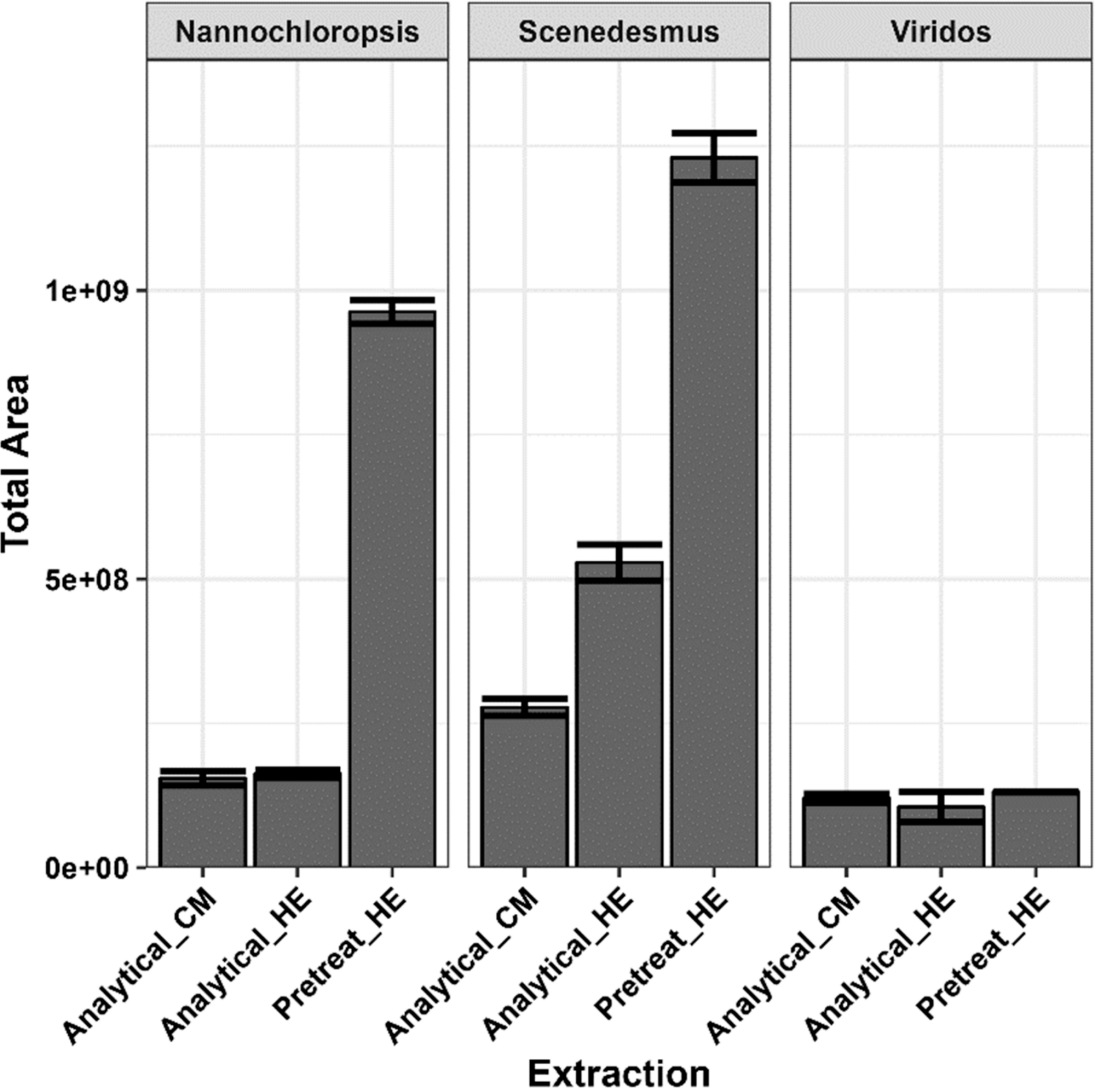
Chlorophylls and chlorophyll-derivatives in extractable oils, shown as the summed area of all associated chromatographic peaks. The information for individual compounds is listed in Table S1.

One of the major organic nitrogen sources in algae oil extracts is chlorophyll compounds and their derivatives. These photosynthetic pigments in their native form consist of a tetrapyrrolic porphyrin structure with a magnesium atom bound to the center with an ester-linked hydrophobic phytol chain. They are related to nickel and vanadium porphyrins that are observed in heavy petroleum oils and have been shown to form coke and deposit metals on hydrodesulfurization catalysts in petroleum refining, leading to catalyst deactivation and poisoning. Although the acid pretreatment resulted in a reduction of polar lipids in the extracted oils, as was observed by lipidomics analysis as well as the percentage of FAME in the extracted oils, we did observe a significant increase in the signal that was attributed to chlorophyll and chlorophyll derivatives in the pretreated oils. However, the trend of an increased porphyrin signal was only observed in the oil extracted from the high-protein biomass. This observation is likely due to the overall higher concentration of chlorophylls naturally produced in nutrient-rich high-protein algae,^21^ as well as lower amounts of other coextracted compounds and lower concentrations of polar head groups, that lead to increased overall concentration in the quantity of chlorophylls present in the oils. There was no significant increase in observed chlorophyll derivatives for the high-lipid Viridos algae. This can be attributed to lower quantities of chlorophyll produced in high-lipid induction, nutrient depleted algae, and the fact that the lipid composition is not greatly modified by acid pretreatment due an almost exclusive presence of TG and DG lipids. Although there was an increase in the overall quantity of chlorophylls, and chlorophyll-derived peaks, in the extracted oil (Figure 3), previous work has shown that bleaching pigments with silica and phosphoric acid can remove chlorophyll structures prior to upgrading.^7^ This work also revealed that chlorophyll structures undergo modifications due to the acid pretreatment that results in demetalation of the porphyrin, removal of hydroxyl functionalities, and in some cases removal of the phytol chain to produce pheophorbide.^10^ The removal of Mg from the porphyrin is beneficial to the overall process as it has been shown that group I and II metals lead to catalyst poisoning of hydrodeoxygenation catalysts in renewable fuels.^19^ Detection and quantification of porphyrin structures in algae oils will certainly be necessary prior to upgrading, and understanding the chemical nature of extracted pigments after acid pretreatment will help to properly identify and quantify the broad range of chlorophyll derivatives that are produced during acid pretreatment. Table S1 shows the chlorophylls and chlorophyll derivatives that were identified by accurate mass and MS/MS analysis along with the peak areas for each compound.

## Conclusions

The demand for a growing and more diverse feedstock portfolio for SAF production is steadily increasing to meet the targets set for 100% sustainable aviation fuel. Algae are projected to contribute as a lipid- and carbohydrate-rich feedstock, based on the ability to leverage two ASTM approved pathways to SAF, ATJ-SPK from fermented carbohydrates and HEFA-SPK from algae lipids, assuming the stringent quality metrics can be achieved and support compatibility with existing processes such as those developed for fats, oils, and greases. This work highlights the potential for utilization of scalable dilute-acid pretreatment to increase both the yield and quality of oil extraction from both high-protein and high-lipid algae. We show that the overall mass yield of extracted oil is lower for acid pretreated biomass when compared to mechanical disruption; however, the oil purity (fatty acid content) of the extracted oils and the overall fatty acid recoveries were significantly higher with acid pretreatment across all three algae species. Acid pretreatment also resulted in an overall reduction in the extraction of polar lipids that contain catalyst poisoning heteroatoms like phosphorus. High-lipid content biomass derived from an engineered algae strain proved to be an ideal feed for conversion to SAF due to the almost exclusive composition of nonpolar, triglyceride lipids, making the extractable oils the closest to a potential drop-in replacement for current HEFA-SPK feeds. Recalcitrant organic nitrogen compounds, *e.g*., porphyrins, were observed and shown to increase the signal observed by mass spectrometry after acid pretreatment. However, the removal of Mg from the porphyrins post-acid pretreatment may improve opportunities for Mg impurity removal strategies, thereby decreasing the risk of catalyst poisoning during hydrodeoxygenation. Understanding the chemical profile of the heteroatom organic origins can help design strategies for removal if they prove to be detrimental to catalyst and process health. Enhanced analytical techniques will be necessary to determine the feasibility of algae oils for hydroprocessing. Overall, the application of dilute acid pretreatment was shown to be a viable biomass pretreatment method, applied prior to lipid extraction, that is scalable and leads to superior oil qualities compared with the oils without pretreatment.

## Supporting Information Available

Supplemental figures for lipidomics, chlorophyll and -derived products m/z features, FAME analysis, and Materials and Methods (PDF)
Mass spectral features identified by MZmine3 (XLSX)
File name key for MZmine3 data (XLSX)
Lipid annotation data from LipidSearch (XLSX)
