# Supplementary material for: High-Resolution Lipidomics Reveals Influence of Biomass and Pretreatment Process on the Composition of Extracted Algae Oils As Feedstock for Sustainable Aviation Fuels
Source: Energy Fuels. 2024 Mar 27;38(7):6547–52. doi: 10.1021/acs.energyfuels.3c04857 (PMC11000214; doi:10.1021/acs.energyfuels.3c04857)
Supplement: Supplementary file 1 — ef3c04857_si_001.pdf [file ef3c04857_si_001.pdf]

Supporting information **DOI: 10.1021/acs.energyfuels.3c04857**

High-Resolution Lipidomics Reveals Influence of Biomass and Pretreatment Process on the Composition of Extracted Algae Oils as Feedstock for Sustainable Aviation Fuels

Steven M. Rowland[1], Stefanie Van Wychen[1], Tao Dong[1], Roger Leach[2], Lieve M. L. Laurens[1*]

[1] Bioenergy Science and Technology Directorate, National Renewable Energy Laboratory, 15013 Denver West Parkway, Golden, CO 80401

[2] Viridos (formerly Synthetic Genomics), 11149 N Torrey Pines Rd, La Jolla, CA 92037

[*]author for correspondence (lieve.laurens@nrel.gov)

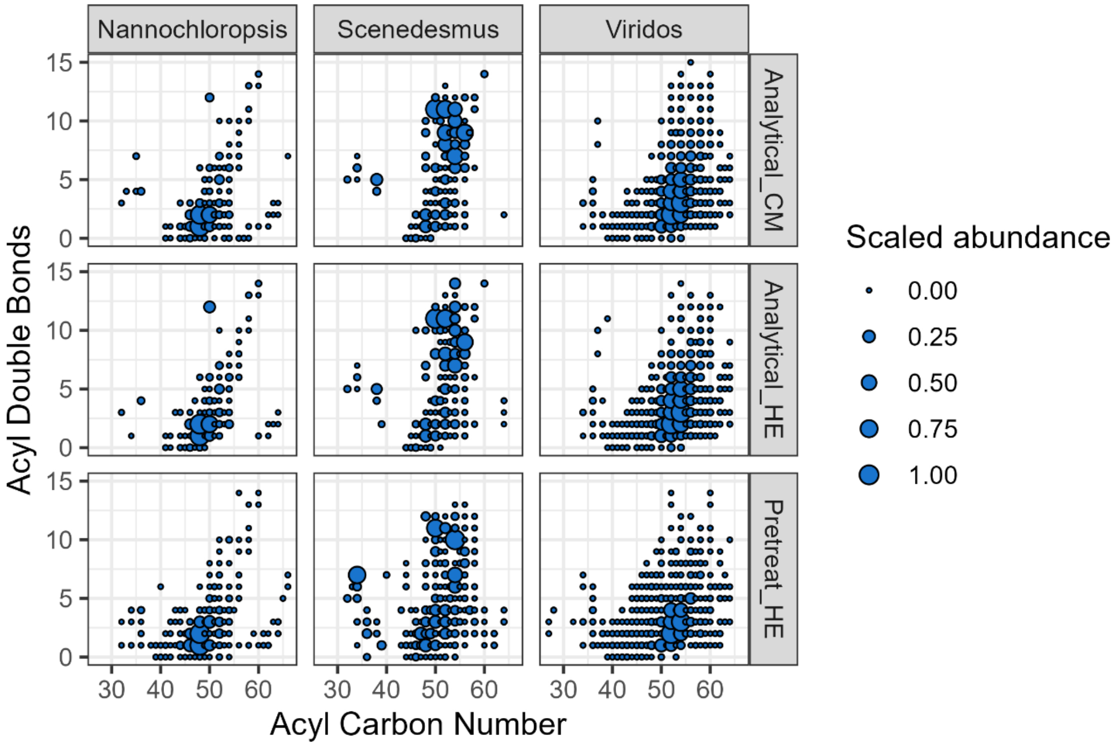

Figure S1. Positive-ion ESI derived plots of acyl double bonds and acyl carbon number that correspond to TG lipids for each algae sample and extraction method. The abundance (represented by point size) was scaled to the most abundant ion for each pane in the figure.

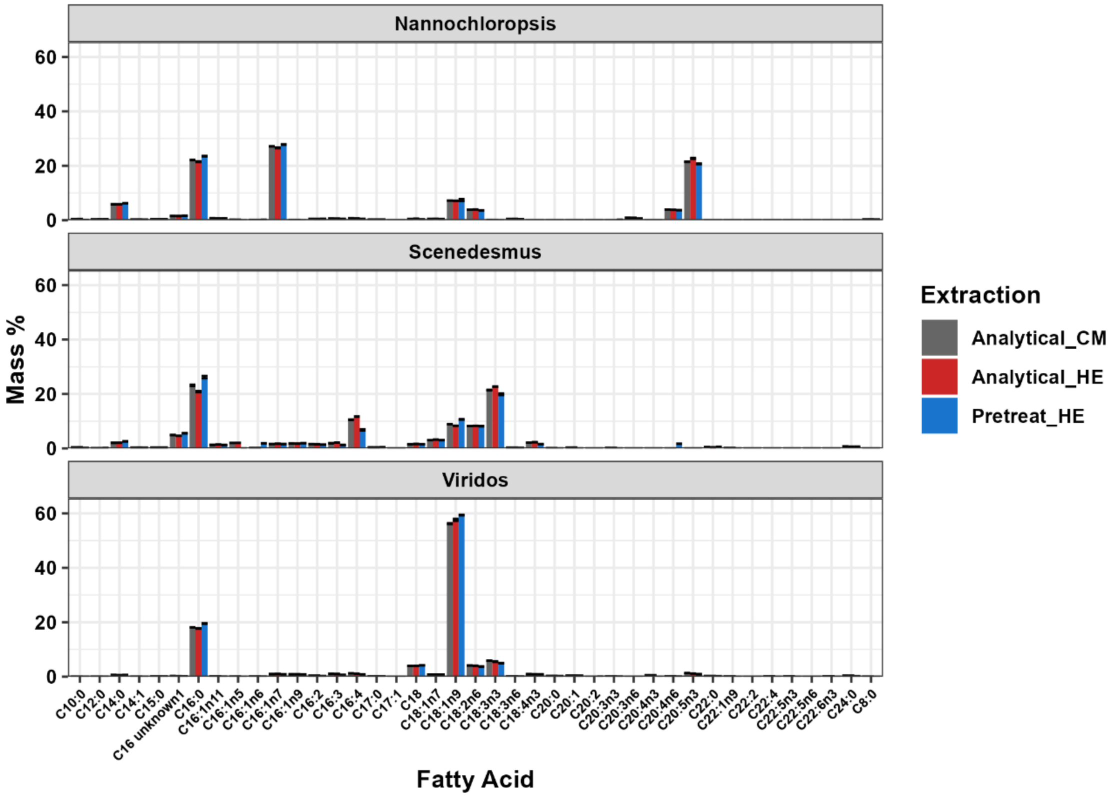

Figure S2: Summary of fatty acid profile for *Nannochloropsis* (top), *Scenedesmus* (middle), and Viridos (bottom) algae with analytical extractions using chloroform:methanol (CM) and hexane:ethanol (HE) mixtures, as well as the dilute acid pretreated extract with hexane:ethanol.

Table S1. Identified chlorophylls and chlorophyll-derived products from dilute acid pretreatment. Abbreviations used: rt = retention time, CM = chloroform:methanol, HE = hexane:ethanol

Chlorophyll Information					*Scenedesmus* sp.			*Nannochloropsis* sp.			Viridos strain #15		
Main ion (m/z)	Chlorophyll type	Formula	rt_ min	rt_ max	Analytical - CM	Analytical - HE	Pretreat - HE	Analytical - CM	Analytical - HE	Pretreat - HE	Analytical - CM	Analytical - HE	Pretreat - HE
533.255	Unknown	C33H32N4O3	1.4	1.65	2.80E+05	4.40E+05	2.70E+06	-	-	3.10E+06	-	-	1.60E+04
535.27	likely pheophorbide	C33H34N4O3	1.7	2	-	-	1.00E+08	-	-	1.20E+08	1.60E+04	3.00E+03	4.30E+06
811.551	Unknown	C53H70N4O3	7.1	7.4	-	-	2.60E+07	-	-	2.70E+07	-	-	2.10E+05
811.551	Unknown	C53H70N4O3	7.41	7.8	-	-	8.70E+06	-	-	1.10E+07	-	-	1.10E+06
813.565	Pyropheophytin a	C53H72N4O3	7.5	7.9	-	2.40E+03	4.30E+08	1.30E+04	-	3.80E+08	4.90E+04	4.10E+03	8.00E+07
815.546	Unknown	C52H70N4O4	6.7	6.9	-	-	2.10E+07	-	-	1.20E+07	-	-	7.60E+05
815.546	Unknown	C52H70N4O4	6.91	7.1	-	-	8.60E+06	-	-	6.70E+06	-	-	7.60E+04
825.531	Unknown	C53H68N4O4	5.95	6.15	-	-	5.10E+05	-	-	2.70E+05	-	-	-
825.531	Unknown	C53H68N4O4	6.35	6.55	-	-	1.80E+06	-	-	2.00E+04	-	-	4.20E+04
825.531	Unknown	C53H68N4O4	6.9	7.15	-	-	1.60E+06	-	-	3.50E+04	-	-	-
827.547	Pyropheophytin b	C53H70N4O4	6.7	7	-	-	1.70E+08	1.60E+03	-	2.30E+06	-	-	1.30E+07
829.525	Unknown	C52H68N4O5	6.1	6.25	-	-	8.20E+06	-	-	8.70E+04	-	-	2.30E+05
831.578	Unknown	C53H74N4O4	6.3	6.6	-	-	1.20E+07	-	-	1.80E+07	-	-	1.60E+06
847.574	Unknown	C53H74N4O5	5.8	6	6.10E+03	1.40E+04	9.20E+06	-	5.10E+03	5.30E+06	-	2.50E+03	2.90E+05
869.558	Unknown	C55H72N4O5	6.7	6.9	1.70E+06	9.60E+06	-	2.80E+04	6.50E+04	-	1.70E+05	1.40E+05	-
871.572	Pheophytin a	C55H74N4O5	7.1	7.3	1.50E+08	3.80E+08	-	8.60E+06	1.40E+07	-	8.40E+07	9.20E+07	-
887.567	Hydroxypheophytin a	C55H74N4O6	6.95	7.15	5.70E+06	1.70E+07	-	3.00E+06	3.30E+06	-	3.60E+07	6.90E+06	-
893.541	Chlorophyll a	C55H72N4O5Mg	6.9	7.05	8.90E+07	7.70E+07	-	9.00E+07	8.50E+07	-	6.50E+04	4.90E+06	-
893.541	Chlorophyll a	C55H72N4O5Mg	7.1	7.25	7.60E+06	1.00E+07	-	5.20E+07	5.80E+07	3.80E+03	-	-	-
907.52	Chlorophyll b	C55H70N4O6Mg	6.1	6.25	2.70E+07	3.20E+07	-	9.60E+05	1.70E+06	-	4.60E+04	1.70E+06	-

Materials and Methods

Materials and biomass sources. All solvents were used as received. Algae material was freeze-dried prior to analysis and obtained from collaborators. Two high-protein algae, *Nannocloropsis sp.* and *Scenedesmus sp.* that has been previously described as the NREL quality control algae samples,[1] and a proprietary strain bioengineered by Viridos Inc. (La Jolla, CA) for high lipid productivity were used for this comparison.

Nannochloropsis and *Scenedesmus* algal biomass was harvested under nutrient replete conditions from the Arizona Center for Algae Technology (AzCATI) at Arizona State University. The Viridos strain was cultivated at the Viridos CAAF (California Advanced Algae Facility) algae R&D site near Calipatria, CA in the Imperial Valley. This cultivation employed a two-stage sequential process where a first stage of nutrient replete growth is followed by a second nutrient deplete "induction phase" to maximize lipid accumulation.[2] All biomass described here was lyophilized prior to analysis to preserve the biomass composition.

Analytical Extraction. The analytical extraction was based on a previous work,[1] Briefly, the freeze-dried algae was subjected to bead beating in 1 minute intervals, for a total of six minutes. The samples were stored on ice between rounds of beadbeating to reduce thermal degradation. Approximately 150 mg of algae was then rehydrated at 4 ºC with 1 mL of ultrapure water for ~12 hr. After rehydration, the samples were centrifuged, and water was carefully decanted ensuring that no biomass was lost. For the chloroform:methanol (C:M) extraction, methanol (volume) was first added and vortexed for 30 s. Chloroform (volume) was then added and vortexed again for 30 s. The samples were centrifuged, and the extract was decanted to pre-weighed vials. For the hexane:ethanol (H:E) extraction, ethanol (volume) was first added and vortexed for 30 s. Hexane (volume) was then added and vortexed again for 30 s. The samples were centrifuged, and the extract was decanted to pre-weighed vials. The solvent was removed under a gentle stream of clean nitrogen at 20 ºC prior to analysis. All samples were subjected to 6 rounds of extraction to ensure the completeness of lipid extraction. Each sample extraction was performed in triplicate.

Pretreatment and Extraction: The dilute acid pretreatment was based on previous work.[3] Freeze-dried biomass samples were added to 10 mL microwave vials, and volume mL water was added. The pH was adjusted with sulfuric acid to a pH of ~4. The samples were vortexed and allowed to sit at room temperature for 30 min. Each sample was heated to 120 ºC in a microwave digester for 10 min. and allowed to cool overnight. A small aliquot of the acid solution (approximately 6 mL) was removed to accommodate the extraction of lipids. A mixture of hexane:ethanol (75:25 v/v) was added and vortexed for 30 s. The samples were then stirred with a magnetic stir plate for 2 hr. with 30 s. of vortexing every 30 min. The organic

phase was then decanted, and this process was repeated 3 times to ensure the lipids were extracted to completeness. The solvent was removed by evaporation under a stream of nitrogen prior to analysis. Each sample was pretreated and extracted in triplicate.

FAME Analysis: The fatty acid content in the biomass and in the extracted oils was determined through the quantification of fatty acid methyl esters (FAME). The FAME analysis on whole biomass was performed as previously described[1,4]. Briefly, approximately 10 mg of the freeze-dried biomass or the equivalent mass of oil was dried under vacuum for 48 hr. at 40 ^{o}C. An internal standard (tridecanoic acid) dissolved in hexane was added to each sample. After addition of the internal standard 0.2 mL of Chloroform:methanol (2:1 v/v) was added, followed by 0.3 mL of methanolic HCL (2.1 % by volume). The samples were heated at 85 ^{o}C for 1 hr. then 1 mL of *n*-hexane was added for FAME extraction. FAME analysis was conducted with gas chromatography coupled to a flame ionization detector (GC-FID) for identification and quantification of known fatty acids. The GC was outfitted with an Agilent 7890 N; DB-WAX-MS column (30 m x 0.25 mm i.d. and 0.25 µm film thickness). The temperature program, flow rates, and standards used for this method have been previously published elsewhere.[1,5]

Lipidomics Analysis: Lipidomics analysis was conducted with a Thermo Scientific Q Exactive mass spectrometer coupled with a Thermo Scientific Vanquish binary liquid chromatograph. Data dependent analysis was conducted for lipidomics with positive-ion electrospray ionization (ESI) with the MS1 scans set to a resolution of 70,000 and an AGC target of 3e6. The scan range was set from m/z 200-2000 to ensure the full range of lipid composition. The top 10 ions from each MS1 scan were selected for MS2 analysis and an ion exclusion list was generated from the method blank to reduce the number of background ions selected for fragmentation. The MS2 conditions were operated at a resolution of 17,500 with an AGC target of 2e5. Dynamic exclusion was set to 20 s to increase the number of MS2 annotations while ensuring that fragmentation of isomers was accomplished. Stepped normalized collision energies of 25 and 30 eV were used for fragmentation of MS2 scans. The ionization parameters were: sheath gas – 45, Aux gas – 8, Sweep gas – 1, electrospray voltage – 3.5 kV, Capillary temperature – 320 ^{o}C, Aux gas heater temp – 375 ^{o}C, and S-lens RF level – 50 V. The separation was performed with a Waters Acquity UPLC BEH C8 column (2.1 x 100 mm and 1.7 µm particles size) with corresponding guard column. The separation was based on work by Cajka and Fiehn[6] and was conducted under gradient conditions with Solvent A consisting of H_2O:ACN (60:40 v/v) and 10 mM ammonium formate and Sovlent B consisting of IPA:ACN (90:10 v/v) with 10 mM ammonium formate. We should note that ammonium formate was not initially soluble in Solvent B so the appropriate amount of ammonium formate was added to 1 mL of water then the solution was added Solvent B. No precipitation was observed with this approach. The separation was performed at 0.4 mL/min, and the gradient is shown in Table S1.

Data analysis was conducted with LipidSearch software. Annotations were made based on a 5 ppm mass error tolerance and limited to fragmentations scores of A or B. Data sorting and plotting was conducted in R programming language. The MS1 data was also processed with MZmine3 software to extract lipid m/z values and chromatographic peak areas. The MZmine 3 data was used for the analysis of chlorophyl-derived pigments in the extracted oils. Pigment annotations were curated manually based on theoretical formula assignment of precursor and fragment ions. The molecular formula calculator tool was used to determine the molecular formula of the observed ions. Table S2 lists the characteristics used to make the pigment annotations. R programming language was used to filter the MZmine3 data and make pigment annotations.

References

(1) Van Wychen, S.; Rowland, S. M.; Lesco, K. C.; Shanta, P. V.; Dong, T.; Laurens, L. M. L. Advanced Mass Balance Characterization and Fractionation of Algal Biomass Composition. *J Appl Phycol* **2021**, *33* (5), 2695–2708. https://doi.org/10.1007/s10811-021-02508-x.

(2) Klein, B.; Davis, R. *Algal Biomass Production via Open Pond Algae Farm Cultivation: 2022 State of Technology and Future Research*; NREL/TP-5100-85661; National Renewable Energy Laboratory: Golden, CO, 2023.

(3) Dong, T.; Van Wychen, S.; Nagle, N.; Pienkos, P. T.; Laurens, L. M. L. Impact of Biochemical Composition on Susceptibility of Algal Biomass to Acid-Catalyzed Pretreatment for Sugar and Lipid Recovery. *Algal Research* **2016**, *18*, 69–77. https://doi.org/10.1016/j.algal.2016.06.004.

(4) Van Wychen, S.; Laurens, L. M. L. *Total Fatty Acid Content Determination of Whole Microalgal Biomass Using In Situ Transesterification*; 2020; Vol. 1980. https://doi.org/10.1007/7651_2017_107.

(5) Laurens, L.; Quinn, M.; Van Wychen, S.; Templeton, D.; Wolfrum, E. J. Accurate and Reliable Quantification of Total Microalgal Fuel Potential as Fatty Acid Methyl Esters by in Situ Transesterification. *Analytical and Bioanalytical chemistry* **2012**, *403* (1), 167–178.

(6) Cajka, T.; Fiehn, O. Increasing Lipidomic Coverage by Selecting Optimal Mobile-Phase Modifiers in LC–MS of Blood Plasma. *Metabolomics* **2016**, *12* (2), 34. https://doi.org/10.1007/s11306-015-0929-x.
